# Morphological Evidence of Telocytes in Skeletal Muscle Interstitium of Exercised and Sedentary Rodents

**DOI:** 10.3390/biomedicines9070807

**Published:** 2021-07-13

**Authors:** Silvia Ravalli, Concetta Federico, Giovanni Lauretta, Salvatore Saccone, Elisabetta Pricoco, Federico Roggio, Michelino Di Rosa, Grazia Maugeri, Giuseppe Musumeci

**Affiliations:** 1 Department of Biomedical and Biotechnological Sciences, Human, Histology and Movement Science Section, University of Catania, Via S. Sofia 87, 95123 Catania, Italy; silviaravalli@gmail.com (S.R.); giovannilau91@hotmail.it (G.L.); elisabettap@tiscali.it (E.P.); federico.roggio@unipa.it (F.R.); mdirosa@unict.it (M.D.R.); graziamaugeri@unict.it (G.M.); 2 Department of Biological, Geological and Environmental Sciences, Section of Animal Biology, University of Catania, Via Androne 81, 95124 Catania, Italy; federico@unict.it (C.F.); saccosal@unict.it (S.S.); 3 Department of Psychology, Educational Science and Human Movement, University of Palermo, Via Giovanni Pascoli 6, 90144 Palermo, Italy; 4 Research Center on Motor Activities (CRAM), University of Catania, Via S. Sofia 97, 95123 Catania, Italy; 5 Department of Biology, College of Science and Technology, Temple University, Philadelphia, PA 19122, USA

**Keywords:** telocytes, skeletal muscle, stem cell niche, sedentary behavior, exercise, CD34, CD117, vimentin

## Abstract

Skeletal muscle atrophy, resulting from states of hypokinesis or immobilization, leads to morphological, metabolic, and functional changes within the muscle tissue, a large variety of which are supported by the stromal cells populating the interstitium. Telocytes represent a recently discovered population of stromal cells, which has been increasingly identified in several human organs and appears to participate in sustaining cross-talk, promoting regenerative mechanisms and supporting differentiation of local stem cell niche. The aim of this morphologic study was to investigate the presence of Telocytes in the tibialis anterior muscle of healthy rats undergoing an endurance training protocol for either 4 weeks or 16 weeks compared to sedentary rats. Histomorphometric analysis of muscle fibers diameter revealed muscle atrophy in sedentary rats. Telocytes were identified by double-positive immunofluorescence staining for CD34/CD117 and CD34/vimentin. The results showed that Telocytes were significantly reduced in sedentary rats at 16 weeks, while rats subjected to regular exercise maintained a stable Telocytes population after 16 weeks. Understanding of the relationship between Telocytes and exercise offers new chances in the field of regenerative medicine, suggesting possible triggers for Telocytes in sarcopenia and other musculoskeletal disorders, promoting adapted physical activity and rehabilitation programmes in clinical practice.

## 1. Introduction

The skeletal muscle is a highly dynamic and plastic tissue that promptly responds to physical activity and sedentary behavior [1]. Skeletal muscle disuse atrophy (SMDA) refers to biochemical, morphological, and functional changes in skeletal muscle that may result from states of hypokinesis or immobilization, e.g., following fractures or elective orthopedic surgery, and represents a major topic in the fields of regenerative and rehabilitation medicine [2,3]. On the contrary, both resistance and aerobic training induce metabolic changes within the muscle by altering protein synthesis, muscle proteolysis, therefore inducing molecular and cellular adaptations that regulate homeostasis and hypertrophy [4,5,6]. A large variety of stromal cells are involved during physiological processes following physical exercise, in order to sustain remodeling and regeneration [7,8]. More specifically, the involvement of Pax+ cells, side population cells (SP cells), pericytes, fibro/adipogenic progenitor cells (FAPs), and PW1+/Pax7– interstitial progenitor cells (PICs), has received attention to investigate exercise-based interventions preventing sarcopenia or in response to injury [8,9,10,11,12,13].

Alexander Mauro described, for the first time, in 1961, a population of mononucleated cells which had been called “satellite cells” (SCs) by virtue of their localization: underneath the surrounding basal lamina and outside the plasma membrane of the muscle fiber they are associated with [14]. These cells have been immunophenotypically identified by Pax7 [15], M-cadherin [16], CD34 [17], and α7-integrin [18] and originate from Pax3+ progenitors in the somites of the embryo that migrate to the limb bud [19,20,21]. In his dissertation, Mauro speculated about the role of these cells to be involved in muscle regeneration as dormant myoblasts, able to initiate the development of skeletal muscle fibers in the event of damage or exogenous triggers [14,22,23]. Besides SCs, other cells concur to the maintenance of skeletal muscle homeostasis and contribute to stem cell niche [24]. Fibroblasts proliferate in close proximity to satellite cells [25], providing structural reliability through assembly of collagen, elastic fibers, and other matrix substances [12,26,27,28]. FAPs are mesenchymal resident cells able to sustain SCs differentiation during tissue regeneration [29,30,31]. Furthermore, muscle-associated vessels accommodate pericytes and mesoangioblasts, participating in endothelial cell communication, angiogenesis, mechanisms of survivals, and cross-talk [32].

**Figure 1:**
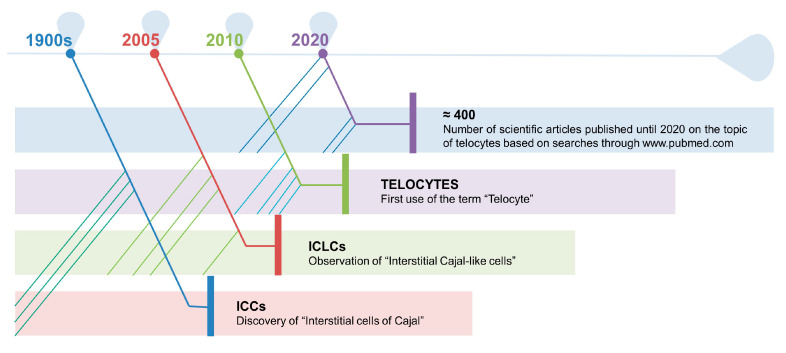
At the beginning of 1900, Santiago Ramón y Cajal described the presence of what he considered primitive interstitial neurons in the loose connective tissue of the tunica muscularis of the gut. Half a century later, M.S. Faussone-Pellegrini and L. Thuneberg observed that these cells were not neurons and called them “Interstitial Cells of Cajal (ICCs)”. These type of cells were then found in many other organs. Finally, Faussone-Pellegrini together with L. M. Popescu, proposed, in 2010, to use the term “Telocyte” to indicate an ICLC. Since their identification, TCs have received attention and the number of scientific articles on the topic is growing considerably.

Lastly, another type of cell, recently identified in the muscle interstitium, is represented by the so-called “Telocytes” (TCs), which appear to physically reside near satellite cells, nerve, and microvascular network [13]. The discovery of this population is referred to as a case of serendipity by the Romanian research group led by Professor Laurentiu M. Popescu, just ten years ago [33]. To understand the road that leads to the definition of these new type of cells, it needs to be reminded the work of Santiago Ramón y Cajal, who described, at the beginning of 1900, the presence of unknown cells in the loose connective tissue of the tunica muscularis of the gut, considering them as primitive interstitial neurons [34]. Although their existence was not fully recognized by the scientific community for half a century, M.S. Faussone-Pellegrini [35] and, independently, L. Thuneberg [36] acknowledged that these cells were not neurons and called them “Interstitial Cells of Cajal (ICCs)”. Extended studies, following the annotation of Cajal, lead to the identification of Interstitial Cajal-like Cells (ICLCs), named “Telocytes” in 2010, in many organs [33], testifying the ubiquity of the novel cell type [37]. Since they were identified, the number of scientific works on TCs is growing exponentially [38] (Figure 1).

It is noteworthy to mention that this discovery raised skepticism and controversy, since ICLCs were also described merely as CD34-positive stromal cells acting as stem cells during regeneration processes [39,40], or the term was used interchangeably to describe fibroblasts [41]. Therefore, there is a need for new evidence that can discriminate the different populations hosted in the stem cell niche of the tissues, not only for their morphology but also for their function. TCs are typically described as cells with small bodies, reported as pear-, spindle-, triangular-shaped, and very long cytoplasmic processes, up to hundreds of micrometers but only approximately 0.2 μm thick [42,43]. The identification of TCs, via transmission electron microscopy, showed characteristic features [33,44] (Table 1). 

Although it is still unclear, the role of TCs seems to participate in sustaining cross-talk communication between stromal cells through signaling transmission via exosomes [13,45], secreting vascular endothelial growth factor (VEGF) and, broadly, promoting myofibers regenerative mechanisms by supporting local stem cell niche differentiation, vasculogenesis, and preventing fibrosis [46]. The presence of this population has been observed, under physiological conditions, in numerous organs and tissues [47,48], and also following pathologic situations, such as musculoskeletal injuries [49], suggesting their role in healing processes and their function for regenerative medicine strategies [50,51,52,53]. As already mentioned, physical inactivity and activity have been extensively studied in relation to stem cell niche and, more recently, are also attracting attention with regard to TCs [49,54,55].

Finally, the aim of this morphologic study was to investigate the presence/absence of TCs in tibialis anterior muscle of healthy rats who underwent a protocol of endurance training for either 4 weeks or 16 weeks in comparison to sedentary rats who were inactive, i.e., not engaging in any physical exercise, throughout the duration of the experiment.

## 2. Materials and Methods

### 2.1. Ethical Approval 

All the procedures involving alive animals were performed at the Center for Advanced Preclinical In Vivo Research (CAPIR), University of Catania. The guidelines of the Institutional Animal Care and Use Committee (I.A.C.U.C.) of the University of Catania (Approved protocol n. 2112015-PR of the 14.01.2015, Italian Ministry of Health) have been complied with. Animal care and handling were carried out in accordance with the EU Directive 2010/63/EU, as well as the Italian law (D.Lgs. 26/2014).

### 2.2. Animals: Housing and Breeding

Two-month-old healthy female Wistar outbred rats, with a bodyweight of 200 ± 20 g, were purchased from Charles River Laboratories, Milan, Italy and bred in the animal facilities at the University of Catania. Rats were maintained and kept in polycarbonate cages (10.25″ W × 18.75″ D × 8″ H) in stable hygrometric and thermic conditions (20–23 °C) on 12 h light/dark cycle with ad libitum access to water and food, throughout the whole period of the experiment. It was used a standard rat chow: carbohydrates (40%), proteins containing all essential amino acids (30%), and lipids (30%). Lipids were a mixture of neutral fatty acids, saturated fatty acids, and unsaturated fatty acids. Diets were provided by Laboratorio Dottori Piccioni, Gessate (Milan), Italy. Twenty rats were used in this study equally divided into two groups, sedentary and undergoing physical exercise, respectively CTRL and PA, sacrificed at two different time points, 4 and 16 weeks: CTRL4W, control sedentary rats sacrificed at 4 weeks; PA4W, rats performing physical exercise sacrificed at 4 weeks; CTRL16W, control sedentary rats sacrificed at 16 weeks; PA16W, rats performing physical exercise sacrificed at 4 weeks. All animals were randomly distributed to groups. All efforts were made to minimize the number of mice, according to the principles of the 3Rs and the resource equation approach, and reduce their suffering [56]. Throughout the whole period of the experiment, the animals were free to move in the cages and their wellness was monitored through objective observation and daily checks (weight, claudication, fur and eyes appearance, consumption of food and water, lethargy) [57]. The animals were sacrificed by carbon dioxide (CO2) overdose, at the established time points. After euthanasia, tibialis anterior muscles were explanted and processed for the planned experiments since the high responsivity of this muscle to exercise [58].

### 2.3. Treadmill Training 

**Figure 2:**
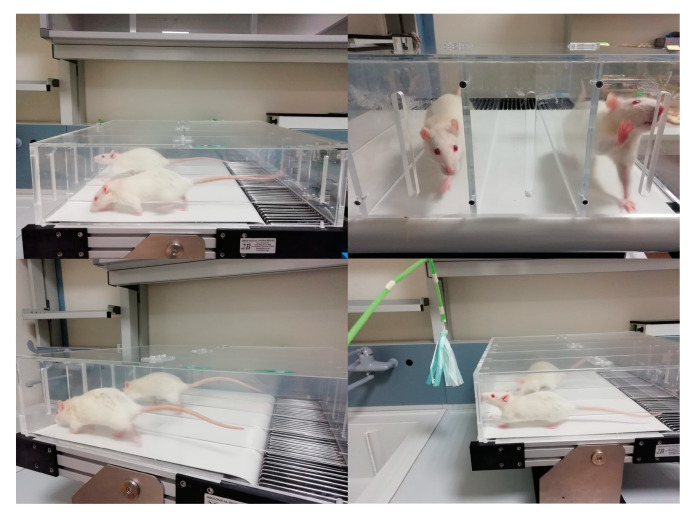
Rats exercising on the treadmill. Speeds and the durations were gradually incremented, respectively, from 10–15 m/min for 5 min to 40–50 m/min for 25 min (from week 1 to week 16).

Two groups of rats (PA4W and PA16W) performed physical activity in the form of running on a treadmill (2Biological instrument, Varese, Italy) (Figure 2 and Supplementary Video S1). Rats were made familiar with the instrument for 1 week prior to surgery, at a speed of 10 m/min (type of exercise: interval training, between mild and moderate) for 5 min daily. This type of exercise is used to stimulate the muscles, joints, and bones in the work of flexion-extension of the limbs. The rats exercised 3 days a week and, in order to adapt the settings to the time-dependent ability of the rats to perform the exercise, the speeds and the durations were gradually incremented from 10–15 m/min for 5 min to 20–30 m/min for 15 min (from week 1 to week 4), from 10–15 m/min for 5 min to 30–40 m/min for 20 min (from week 1 to week 8), from 10–15 m/min for 5 min to 40–50 m/min for 25 min (from week 1 to week 16). The treadmill was gradually inclined between 2° and 6° degrees. A minimal electric shock (0.2 mA) was used to avoid the rat to stop running, if distracted, to stimulate the walking and to instruct the rats in the first place. All-electric shock bouts were closely monitored in real-time and acquired by the embedded data acquisition software (2Biological instrument, Varese, Italy). Rats that exceeded the number of five electric shocks, in one session, were suspended from the exercise.

### 2.4. Histology Analysis

Tibialis anterior muscle samples were washed in phosphate-buffered saline (PBS, Bio-Optica, Milano, Italy), fixed in 10% buffered-formalin (Bio-Optica, Milan, Italy) for 24 h at room temperature. Afterwards, the samples were dehydrated in graded ethanol (Bio-Optica, Milan, Italy), cleaned in xylene (Bio-Optica, Milan, Italy) and paraffin-embedded (Bio-Optica, Milan, Italy), being careful to preserve the desired anatomical orientation. Slides of 5 μm thickness were cut from the obtained paraffin blocks and hematoxylin and eosin-stained (H&E, Bio-Optica, Milan, Italy) following a protocol described elsewhere [59]. The samples were then examined in triplicate for morphological evaluation with a Zeiss Axioplan light microscope (Carl Zeiss, Oberkochen, Germany) and by a digital camera (AxioCam MRc5, Carl Zeiss, Oberkochen, Germany), used to take images.

### 2.5. Histomorphometric Analysis

Each H&E stained muscle cross section was subjected in triplicate to morphometric analysis by calculating the area of twenty muscle fibers of five randomly selected fields with a total area of about 35.000 μm^2^, using a software for image acquisition (AxioVision Release 4.8.2—SP2 Software, Carl Zeiss Microscopy GmbH, Jena, Germany) [60]. Data were then expressed as diameter mean ± standard deviation (SD). Statistical significance of results was thus accomplished. Three investigators (two anatomical morphologists and one histologist) made the morphological assessment. If disputes occurred, a unanimous agreement was reached after section re-evaluation and before proceeding with data interpretation.

### 2.6. Double Immunofluorescence Analysis

Paraffin-embedded muscle tissue sections of 5 μm thickness were subjected in triplicate to double immunofluorescence (IF) combining anti-mouse and anti-rabbit goat secondary antibodies with either mouse or rabbit primary antibodies. Muscle sections were deparaffinized with xylene and rehydrated in graded ethanol scale. Afterwards, the slides were cleaned for 20 min with phosphate-buffered saline (PBS; Bio-Optica, Milan, Italy) and unmasked in citrate buffer (pH 6.0; Bio-Optica, Milan, Italy), or in ethylenediaminetetraacetic acid-Tris buffer (Tris-EDTA pH 8.0, Bio-Optica, Milan, Italy) for the antigenic retrieval and incubated in 0.3% H_2_O_2_/PBS, for 30 min, to block endogenous peroxidase activity. Non-specific antibody binding sites were blocked by applying a solution of 1% bovine serum albumin (BSA; Sigma-Aldrich, Saint Louis, MO, USA) in PBS 1X for 1 h at room temperature. Tissue slides were washed in PBS and, then, incubated overnight at +4 °C with a mixture of mouse and rabbit primary antibodies at appropriate dilution in antibody dilution buffer: mouse monoclonal anti-CD34 (1:100; Dako, Agilent, Santa Clara, CA, USA), rabbit monoclonal anti-CD34 (1:100; Invitrogen, Thermo Fisher Scientific, Waltham, MA, USA), rabbit polyclonal anti-CD117 (1:500; Dako, Agilent, Santa Clara, CA, USA), mouse monoclonal anti-Vimentin (VIM, 1:200; Dako, Agilent, Santa Clara, CA, USA). Primary antibodies were revealed using specific fluorescent-dye Goat anti-Mouse Alexa Fluor 488-conjugated IgG (1 μg/mL; Invitrogen, Thermo Fischer Scientific, Waltham, MA, USA) and Goat anti-Rabbit 594-conjugated IgG (2 μg/mL; Invitrogen, Thermo Fisher Scientific, Waltham, MA, USA) for 1 h at room temperature. Details on primary and secondary antibody sources and dilutions are shown in Table 2. Negative controls were performed by replacing primary antibodies with non-immune serum, while cross reactivity of secondary antibodies was verified by omitting primary antibodies. Immunolabeled samples were rinsed in PBS and mounted using an anti-fade mounting medium containing 4′,6-diamidino-2-phenylindole (DAPI) for nuclear counterstaining (Vectashield, Vector Laboratories, Burlingame, CA, USA) and sealed with nail polish.

### 2.7. Computerized Densitometric Measurements and Image Analysis

Digital micrographs of double immunofluorescence sections were taken using a confocal laser scanning microscopy (CLSM, Zeiss LSM700, Carl Zeiss, Oberkochen, Germany) with ZEN-2010 software. In order to detect the fluorophore signal, three lasers with 405, 488, and 555 nm wavelengths were used for the analysis of blue, green, and red signals, respectively. Image analysis software (AxioVision Release 4.8.2-SP2 Software, Carl Zeiss Microscopy GmbH, Jena, Germany), which quantifies the level of double positive staining of anti-CD34/anti-CD117 and anti-CD34/anti-Vimentin immunolabeling, was used to calculate the densitometric count in five fields, 10× magnification, randomly selected from each section. Statistical results are expressed as densitometric count (pixel^2^)/(pixel^2^) of double immunostaining on muscle tissue. Three blinded investigators (two anatomical morphologists and one histologist) made the evaluations that were assumed to be correct if values have not statistically significant difference. If disputes concerning interpretation occurred, unanimous agreement was reached after sample re-evaluation.

### 2.8. Immunohistochemistry

Skeletal muscle samples, 5 μm-thick, were processed for immunohistochemical analysis. Briefly, the slides were dewaxed in xylene, hydrated using graded ethanols, and therefore heated (5 min × 3) in capped polypropylene slide-holders with citrate buffer—pH 6 (Bio-Optica, Milan, Italy), using a microwave oven (750 W, LG Electronics Italia S.p.A., Milan, Italy) to unmask antigenic sites. The slides were incubated for 30 min in 0.3% H_2_O_2_/PBS to quench endogenous peroxidase activity before being rinsed for 20 min with PBS (Bio-Optica, Milan, Italy). After blocking, the sections were incubated overnight at 4 °C with rabbit monoclonal anti-CD34 (1:100; Invitrogen, Thermo Fisher Scientific, Waltham, MA USA). Immune complexes were then treated with biotinylated link antibodies (horseradish peroxidase polymer (HRP)-conjugated anti-rabbit and anti-mouse were used as secondary antibodies) and then detected with peroxidase-labelled streptavidin, both incubated for 10 min at room temperature (LSAB + System-HRP, K0690, Dako, Glostrup, Denmark). Immunoreactivity was visualized by incubating the sections for 2 min in 0.1% 3,3′-diaminobenzidine (DAB) (DAB substrate Chromogen System, Dako, Glostrup, Denmark). The sections were lightly counterstained with Mayer’s hematoxylin (Histolab Products AB, Göteborg, Sweden), mounted in Glycerol Vinyl Alcohol (GVA) (Zymed Laboratories, San Francisco, CA, USA), observed with an Axioplan Zeiss light microscope (Carl Zeiss, Oberkochen, Germany), and photographed with a digital camera (AxioCam MRc5, Carl Zeiss, Oberkochen, Germany).

### 2.9. Statistical Analysis

Statistical analysis was performed using GraphPad Instat^®^ Biostatistics version 8.0 software (GraphPad Software, Inc., La Jolla, CA, USA). The sample size calculation for this study was established using the resource equation approach, including minimum and maximum sample sizes, because it was not possible to assume standard deviation and effect size [56]. Data were tested for normality with the Kolmogorov–Smirnov and Shapiro–Wilk test. All variables were normally distributed. Differences between experimental groups were evaluated by using one-way ANOVA (histomorphometric and immunofluorescence analysis) and two-way ANOVA (weights) followed by Tukey’s multiple comparison post hoc test. For all experiments, *p*-values of less than 0.05 (*p* < 0.05) were considered statistically significant; p values of less than 0.01 (*p* < 0.01) were considered to be highly statistically significant. The data are presented as the mean value ± SD. Cohen’s κ was applied to measure the agreement between the three blinded observers and averaged to evaluate overall agreement.

## 3. Results

### 3.1. Body Weight

**Figure 3:**
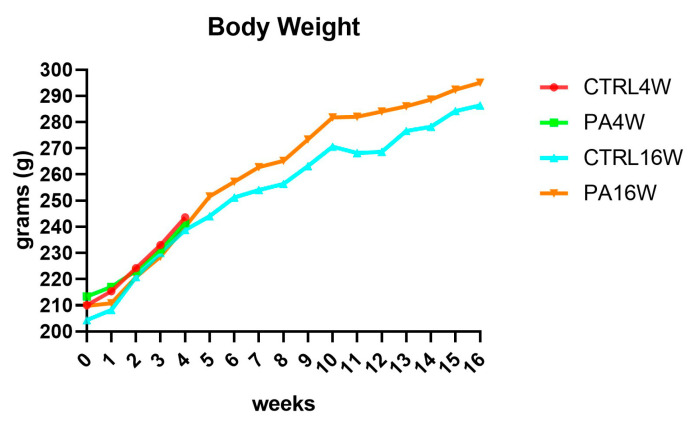
Body weight variations over 16 weeks, showing a physiological increase in all groups. The differences between groups, analyzed by two-way ANOVA followed by Tukey’s multiple comparison post hoc test, are not significant, as expected (*p* > 0.05). CTRL4W, control sedentary rats sacrificed at 4 weeks; PA4W, rats performing physical exercise sacrificed at 4 weeks; CTRL16W, control sedentary rats sacrificed at 16 weeks; PA16W, rats performing physical exercise sacrificed at 4 weeks.

Body weights and food and drink consumption were monitored throughout the experiment, 3 days per week, for a total of 48 time points. A physiological increase in body weight during the weeks in all groups was observed since the differences between groups, for each time point, are never significant (*p* > 0.05), as expected (Figure 3). At the start of the experiment, the mean ± SD body weight of all rats was 209.4 ± 13.52 g, at the end of the fourth week, it was 240.95 ± 10.95 g, reaching 290.7 ± 15.97 g, at the end of the sixteenth week, for the remaining animals.

### 3.2. Histology and Histomorphometry

**Figure 4:**
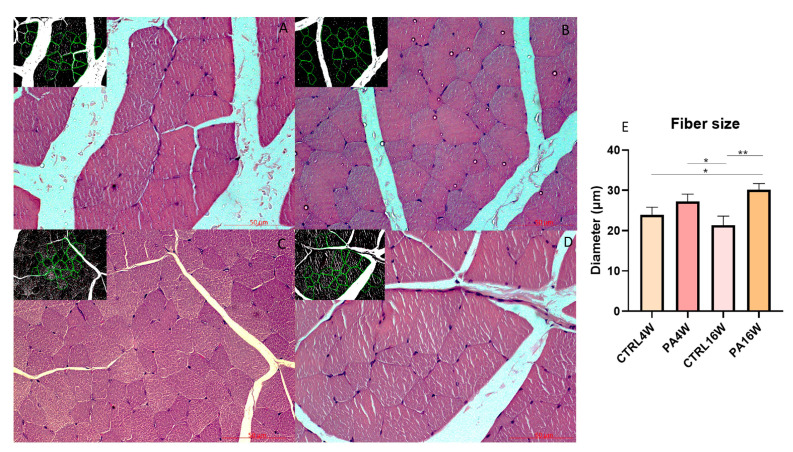
Hematoxylin and eosin staining (**A**–**D**) and morphometric analysis of the diameter (μm) (mean ± SD) of the muscle fibers (**E**). (**A**–**D**) (**A**) group CTRL4W and in the inset morphometric analysis by the software; (**B**) group PA4W and in the inset morphometric analysis by the software; (**C**) group CTRL16W and in the inset morphometric analysis by the software; (**D**) group PA16W and in the inset morphometric analysis by the software. (**E**) The morphometric analysis highlights a significant hypertrophy of the groups PA16W (30.46 ± 1.43 μm) (** *p* < 0.0001), and to PA4W (* *p* < 0.001) when compared to CTRL16W (21.39 ± 2.19 μm). Comparison between CTRL4W and PA16W highlights a significant hypertrophy of the latter group (* *p* < 0.001). No other comparisons show to be significant. Data were tested for normality with the Kolmogorov–Smirnov and Shapiro–Wilk test, and differences between experimental groups were evaluated by using one-way ANOVA, followed by Tukey’s multiple comparison post hoc test. Lens magnification: ×20. Scale bars: 50 μm. ** *p* < 0.0001, * *p* < 0.001. CTRL4W, control sedentary rats sacrificed at 4 weeks; PA4W, rats performing physical exercise sacrificed at 4 weeks; CTRL16W, control sedentary rats sacrificed at 16 weeks; PA16W, rats performing physical exercise sacrificed at 4 weeks.

Histological analysis with H&E were examined to highlight the possible structural alterations in muscle tissue of all experimental groups. No cytological alteration is detected in the muscle fibers of all groups. The morphometric analysis of the diameter (μm) (mean ± SD) of the muscle fibers highlights a significant hypertrophy of the groups PA16W (30.46 ± 1.43 μm) (** *p* < 0.0001) and PA4W (27.47 ± 1.61 μm) (* *p* < 0.001) vs. CTRL16W (21.39 ± 2.19 μm). PA16W (27.47 ± 1.61 μm) also shows a predictable hypertrophy when compared to CTRL4W (24.01 ± 1.57 μm), (* *p* < 0.001). On the contrary, PA16W (30.46 ± 1.43 μm) does not show a statistically significant hypertrophy when compared with group PA4W (27.47 ± 1.61 μm), (ns) (Figure 4). No significant differences are revealed when compared CTRL4W vs. CTRL16W and PA4W vs. CTRL4W.

### 3.3. Double Immunofluorescence and Densitometric Analysis

**Figure 5:**
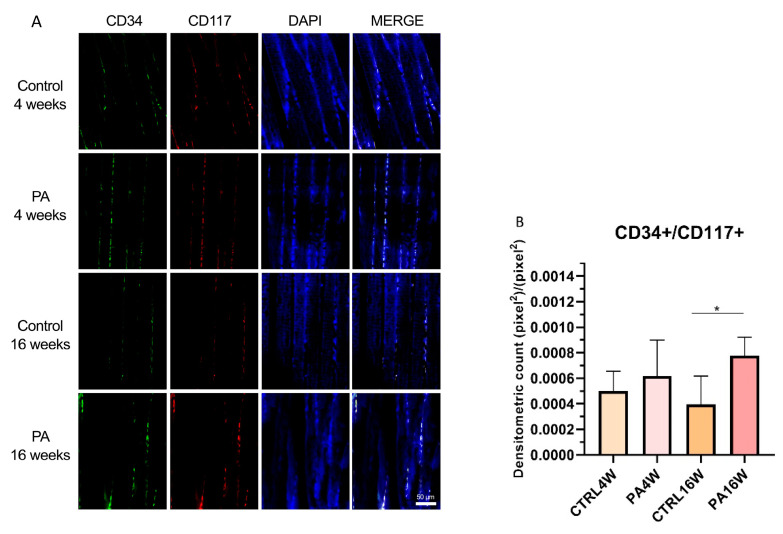
(**A**) Representative images of double immunofluorescence staining for muscle tissue TCs as determined by CD34+/CD117+. CD34 (green) and CD117 (red) immunostaining with 4′,6-diamidino-2-phenylindole (DAPI; blue) counterstain for nuclei. (**B**) Comparison between PA16W and CTRL16W highlights a significant higher expression of TCs in exercised rats at 16 weeks (* p < 0.05). No other comparisons show to be significant. Data were tested for normality with the Kolmogorov–Smirnov and Shapiro–Wilk test, and differences between experimental groups were evaluated by using one-way ANOVA, followed by Tukey’s multiple comparison post hoc test. The slides are scanned by confocal laser scanning microscopy (CLSM; Zeiss LSM700, Oberkochen, Germany) at 200× magnification. Scale bars: 50 μm. CTRL4W, control sedentary rats sacrificed at 4 weeks; PA4W, rats performing physical exercise sacrificed at 4 weeks; CTRL16W, control sedentary rats sacrificed at 16 weeks; PA16W, rats performing physical exercise sacrificed at 4 weeks.

**Figure 6:**
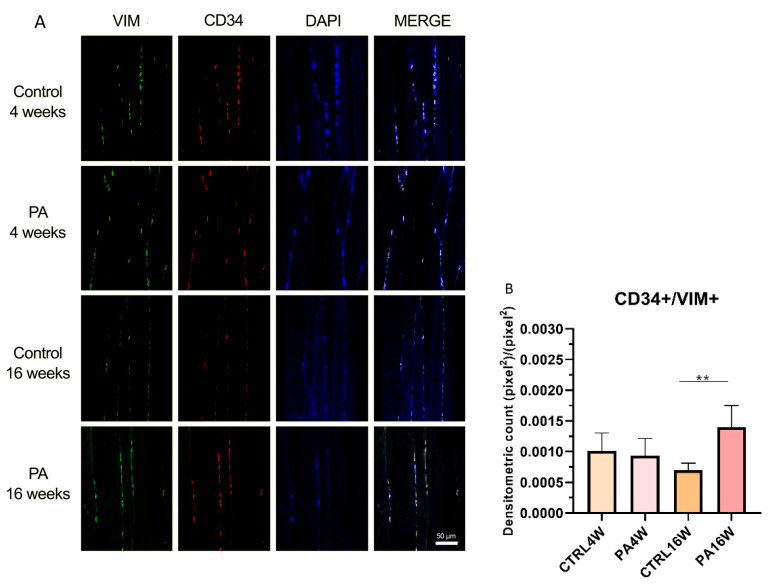
(**A**) Representative images of double immunofluorescence staining for muscle tissue TCs as determined by CD34+/VIM+. CD34 (red) and VIM (green) immunostaining with 4′,6-diamidino-2-phenylindole (DAPI; blue) counterstain for nuclei. (**B**) Comparison between PA16W and CTRL16W highlights a highly significant higher expression of TCs in exercised rats at 16 weeks (** p < 0.01). No other comparisons show to be significant. Data were tested for normality with the Kolmogorov–Smirnov and Shapiro–Wilk test, and differences between experimental groups were evaluated by using one-way ANOVA, followed by Tukey’s multiple comparison post hoc test. The slides are scanned by confocal laser scanning microscopy (CLSM; Zeiss LSM700, Oberkochen, Germany) at 200× magnification. Scale bars: 50 μm. CTRL4W, control sedentary rats sacrificed at 4 weeks; PA4W, rats performing physical exercise sacrificed at 4 weeks; CTRL16W, control sedentary rats sacrificed at 16 weeks; PA16W, rats performing physical exercise sacrificed at 4 weeks.

Although transmission electron microscopy (TEM) examination is the golden standard for TCs identification [33,61], double-immunostaining is currently the most common tool for semi-quantitative analysis of TCs [62,63,64], since it can help in discriminate this population from other interstitial cells. In this work, double positive immunofluorescences for CD34/CD177 and CD34/VIM were used to identify TCs in sedentary and exercised muscle rat tissue at 4 and 16 weeks (Figure 5A and Figure 6A). Statistical results are expressed as mean ± SD of the densitometric count (pixel^2^)/(pixel^2^) of double immunostaining on muscle tissue. CD34 and CD117 double labeling analysis indicates a statistically significant increase in the expression of TCs in PA16W (7.7 × 10^−4^ ± 1.3 × 10^−4^ (pixel^2^)/(pixel^2^)) vs. CTRL16W (3.9 × 10^−4^ ± 2 × 10^−4^ (pixel^2^)/(pixel^2^)) (* *p* < 0.05) (Figure 5B). Densitometric values for CTRL4W and PA4W groups are, respectively, 4.9 × 10^−4^ ± 1.4 × 10^−4^ (pixel^2^)/(pixel^2^) and 6.1 × 10^−4^ ± 2.5 × 10^−4^ (pixel^2^)/(pixel^2^). Similarly, the number of interstitial TCs is highly significant higher in PA16W (1.3 × 10^−3^ ± 3.1 × 10^−4^ (pixel^2^)/(pixel^2^)), vs. CTRL16W (6.9 × 10^−4^ ± 1 × 10^−4^ (pixel^2^)/(pixel^2^)) (** *p* < 0.01) as determined by CD34/VIM double-immunostaining (Figure 6B). Densitometric values for CTRL4W and PA4W groups are, respectively, 1 × 10^−3^ ± 2.6 × 10^−4^ (pixel^2^)/(pixel^2^) and 9.3 × 10^−4^ ± 2.5 × 10^−4^ (pixel^2^)/(pixel^2^). No other statistically significant differences are highlighted between groups, in both experiments.

### 3.4. Immunohistochemistry

**Figure 7:**
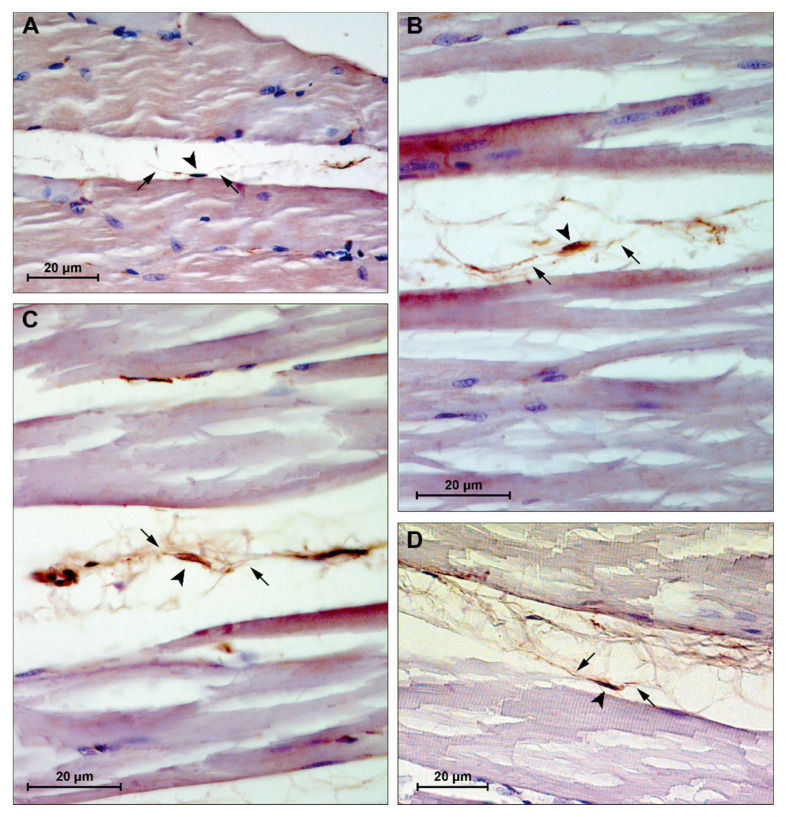
Representative images of immunohistochemistry staining for muscle tissue CD34+ cells. (**A**) group CTRL4W, estimated size body (arrowhead): 5.23 μm, estimated cytoplasmic processes length (arrows): 13.28 and 24.28 μm; (**B**) group PA4W, estimated size body (arrowhead): 5.04 μm, estimated cytoplasmic processes length (arrows): 26.68 and 11.94 μm; (**C**) group CTRL16W, estimated size body (arrowhead): 8.69 μm, estimated cytoplasmic processes length (arrows): 13.07 and 20.56 μm; (**D**) group PA16W, estimated size body (arrowhead): 9.56 μm, estimated cytoplasmic processes length (arrows): 22.03 and 13.36 μm. Lens magnification: ×40. Scale bars: 20 μm. CD34+ cells nuclei and cytoplasmic processes were measured using a caliper tool of the software for image acquisition (AxioVision Release 4.8.2—SP2 Software, Carl Zeiss Microscopy GmbH, Jena, Germany). CTRL4W, control sedentary rats sacrificed at 4 weeks; PA4W, rats performing physical exercise sacrificed at 4 weeks; CTRL16W, control sedentary rats sacrificed at 16 weeks; PA16W, rats performing physical exercise sacrificed at 4 weeks.

Skeletal muscle sections showed CD34+ cells at the periphery of the fibers, within the interstitium between muscle fibers (Figure 7). These cells appear to exhibit morphological features of TCs, i.e., spindle nuclei, approximately 5–10 μm in diameter, and multiple long cytoplasmic processes, approximately 10–25 μm in length and 0.1–0.2 μm in thickness, identifiable with telopodes.

## 4. Discussion

TCs have been largely identified, over the last ten years, as populating the stromal compartments of a variety of organs, belonging to the tissue stem cell niche [33,49,65]. Within the skeletal muscle tissue, TCs are distributed throughout the perimysium and endomysium and could reach long-distances through their telopodes which allow these cells to make contact with myofibers, nerve terminals, blood vessels, and other stromal populations, including SCs, sited beneath the surrounding basal lamina of the myofiber.

In the present morphological study, the presence of TCs in tibialis anterior muscle of healthy rats who underwent a protocol of endurance training for either 4 weeks or 16 weeks was investigate in relation to sedentary rats who were inactive, i.e., not engaging in any physical exercise, throughout the duration of the experiment.

H&E staining shows no cytological alteration in the muscle tissue of all groups, although the morphometric analysis of the size of the muscle fibers highlights a significant atrophy, defined as a decrease in the size of myofibers, of the sedentary control group at 16 weeks (CTRL16W) when compared to physically active rats undergoing treadmill training for both 16 weeks (** *p* < 0.0001) and 4 weeks (* *p* < 0.001) (Figure 4). Muscle morphological adaptations to active/inactive styles were not, however, accompanied by statistically significant differences (*p* > 0.05) in body weights in all groups, indicating a physiological growth of the rats during the weeks, as expected (Figure 3). 

Whether atrophy may affect muscle stem cells numbers or behavior is still controversial and should be further elucidated to assess the role of daily mechanical stress administered through exercise [66]. Mitchell et al. [67] reported that the number of stem cells in hindlimb muscles of mice was reduced after 2 weeks of hind limb suspension, and Verdijk et al. [68] observed similar decline in human vastus lateralis muscle following sarcopenia, reversed by resistance training which increased satellite cell content and type II muscle fiber size. Conversely, aerobic and resistance training have been reported to be a stimulus for the formation of new muscle fibers and maintain their homeostasis [4,5].

Although SCs are the main characters of renewal programme in skeletal muscle, other tissue residents and recruited stromal cells, e.g., fibroblasts, fibro-adipogenic progenitors, endothelial cells, perycites, and macrophages, are paramount supporting players [16,22,69]. Among these cell-cell interactions, TCs and SCs seem to interact by juxtacrine and paracrine intercellular signaling, in order to support in a concerted manner [13] the network mediating new tissue organization [70]. However, the interplay between these two cell types, especially in skeletal muscle injury [49], has yet to be elucidated in depth since cue-based investigations rely mainly on knowledge of the close proximity of TCs to SCs and their ability to communicate. 

This study provides a novel finding that interstitial TCs are decreased in the muscles of sedentary rats compared to exercised rats, supporting the recent reports that TCs are, otherwise, increased in exercise-induced cardiac growth [55], participate in early moderate exercise-induced remodeling after acute myocardial infarct [71] and in eccentric contraction-induced skeletal muscle injury, in rodents. Since their discovery is relatively recent, TCs are not yet defined by specific antigenic markers, although CD34 is the most commonly used antigen to characterize their presence [48,72]. Vimentin, CD117/c-kit, PDGFR-β (platelet-derived growth factor receptor β), and SMA (smooth muscle actin) are the antigens most frequently associated with TCs [47,62,73,74]. Bei et al. analyzed immunofluorescence double staining for cardiac TCs and fibroblasts in vitro. CD34/CD117, CD34/vimentin, and CD34/PDGFR-β were positive for TCs, whereas fibroblasts showed positivity only for vimentin and PDGFR-β [75]. 

In this work, double positivity for CD34+/CD117+ and CD34+/vimentin+ were used to individuate TCs and to discriminate this population from fibroblasts which are CD34-/CD117-/vimentin+. CD34 is a sialomucin mainly expressed in hematopoietic stem cells (HSCs) surface but it has also been found in other tissue-specific stem cells [76]. Vimentin is cytoskeletal type III intermediate filament protein found in mesenchymal-derived cells that provides an architectural network for organelles anchoring to cytoplasm [73]. CD117 is a transmembrane receptor tyrosine kinase widely used for TCs identification which is active in proliferation and differentiation [77]. Based on two distinct double-immunostainings for CD34/CD117 and CD34/Vimentin, skeletal muscle TCs were identified in all groups. CD34/CD177 statistical analysis indicates a significant difference in the higher level of TCs in exercised rats at 16 weeks in relation to their control group (* *p* < 0.05) (Figure 5). This result is also more significantly supported by CD34/VIM double-immunostaining (** *p* < 0.01) (Figure 6). These results indicate a potential targeting of TCs, as belonging to muscle stem cell niche, in cell dysfunction associated with atrophic condition [67]. This latter negatively affects stem cells via presence of catabolic factors such as myostatin [78] and tumor necrosis factor α [79] as well as decreases in trophic factors [80]. In contrast, in this work, rats subjected to regular physical training for 16 weeks maintained a stable TCs population, although not statistically increased compared to rats who performed exercise for only 4 weeks.

Finally, immunohistochemistry showed CD34+ cells, within the interstitium between muscle fibers (Figure 7), with characteristic features of TCs and telopodes, i.e., spindle nuclei and multiple long cytoplasmic processes. These observations are in support with current evidence suggesting TCs’ role in intercellular signaling through their strategic position and organized network of telopodes with local cellular neighborhood, nerves, and capillaries [33,81,82]. Homo- and heterocellular communication seems to be carried out via small molecules and shedding microvesicles carrying various molecules like proteins, RNAs, and microRNA [49,83,84]. These mechanisms of transmission allow rapid cell engagement in the tissue milieu, in order to approach adaptations to biochemical and physical changes. The use of TEM would have been useful to determine the ultrastructural features of TCs in exercised rats and this should be noticed as a limitation of this study.

## 5. Conclusions

These findings herein are intended to encourage knowledge about TCs population and their role in the stem cells niche of skeletal muscles. Further studies investigating TCs in response to different types of exercise (resistance, aerobic, isotonic, flexibility), sedentary behavior, ageing, and pathophysiological conditions, as well as studies on the possibility of triggering TCs through exercise to reverse atrophic conditions, would be scientifically valuable. 

This study may be framed in a field still in its infancy, although it is rapidly attracting the attention of the scientific community. The understanding of the above-mentioned mechanisms between the cells involved in the tissue remodeling process could offer new chances in regenerative tissue strategies and insights about finding possible triggers for TCs in sarcopenia and other musculoskeletal disorders, in clinical medicine.

Finally, since exercise training has been shown to exert protective effects against sedentary-induced atrophy, rather by sustaining muscle remodeling and maintenance of TCs, this finding might promote further skeletal muscle adapted physical activity and rehabilitation programmes for humans.

## Figures and Tables

**Table 1:** Characteristic features of TCs and of their telopodes.

Cell Structure	Characteristic Features of Telocytes	
Body	small, oval- pear- spindle- triangular-shaped; average dimensions: 9.39 μm ± 3.26 μm; the nucleus occupies about 25% of the cell volume and contains clusters of heterochromatin attached to the nuclear envelope	
Cytoplasm	mitochondria: approximately 5%–10% of the cytoplasmic volume; small Golgi complex; endoplasmic reticulum: 1%–2% of the cyto-plasmic volume	
Plasmalemma	thin or absent basal lamina; caveolae occupy about 2–3% of cytoplasmic volume;	
Telopodes	Number	on average from 1 to 5;
	Length	up to hundreds of μm;
	Thickness	uneven calibre, mostly below 0.2 μm;
	Aspect	moniliform with dilations and branches;
	Organization	three-dimensional network communicating through gap junctions;

**Table 2:** Primary and secondary antibody used in IF and their dilutions.

Primary Antibody	Host Species	Producer	Dilution	Secondary Antibody	Producer	Dilution
Anti-CD34	MOUSE	Dako	1:100	AF488	Invitrogen	1 μg/mL
Anti-CD117	RABBIT	Dako	1:500	AF594	Invitrogen	2 μg/mL
Anti-CD34	RABBIT	Invitrogen	1:100	AF594	Invitrogen	2 μg/mL
Anti-VIM	MOUSE	Dako	1:200	AF488	Invitrogen	1 μg/mL

## Data Availability

The data presented in this study are available on request from the corresponding author.

## Supplementary Materials

The following are available online at https://www.mdpi.com/article/10.3390/biomedicines9070807/s1, Video S1: Treadmill training.
